# Keratoacanthoma-Type Invasive Squamous Cell Carcinoma Managed Non-surgically With Topical Immunomodulators

**DOI:** 10.7759/cureus.86267

**Published:** 2025-06-18

**Authors:** Brian A Moreno, Moises Lutwak, Stanley Skopit

**Affiliations:** 1 Dermatology, Lake Erie College of Osteopathic Medicine, Bradenton, USA; 2 Dermatology, Larkin Community Hospital, South Miami, USA

**Keywords:** case report dermatology, clinical dermatology, dermatology care, dermatology oncology, dermatology treatments, dermatology trends, general dermatology, medical dermatology, preventive dermatology, skin disease/dermatology

## Abstract

A rapidly growing lesion with keratoacanthoma-like features may complicate early diagnosis of squamous cell carcinoma (SCC). While most cutaneous SCCs exhibit slow, indolent growth, lesions with keratoacanthoma-like features may demonstrate more rapid expansion, occasionally complicating early diagnosis. We report a case of a 71-year-old male patient with a history of actinic keratoses and non-melanoma skin cancer who presented with a new blistering, erythematous eruption on the left dorsal forearm. A potassium hydroxide (KOH) prep revealed branching hyphae, and the patient was initially treated with oral antifungals. However, biopsy of the lesion confirmed a well-differentiated invasive SCC of keratoacanthoma type. Given the lesion’s size and clinical behavior, the patient was referred for oncology-directed care and successfully treated with topical immunomodulation. PET imaging ruled out metastasis, and clinical resolution was observed on follow-up. This case underscores the diagnostic complexity of keratoacanthoma-like SCCs and highlights the need for biopsy in suspicious or atypical presentations. It also reflects evolving treatment approaches in select cases of cutaneous SCC, particularly among elderly patients with comorbidities or those who decline surgical management.

## Introduction

Cutaneous squamous cell carcinoma (SCC) is the second most common form of skin cancer worldwide and accounts for approximately 20% of all non-melanoma skin cancers [1]. The incidence of SCC continues to rise with an aging population, increased cumulative ultraviolet (UV) exposure, and broader use of immunosuppressive therapies [2]. While most cutaneous SCCs arise in chronically sun-exposed areas and follow a relatively indolent course, certain histologic variants, such as those with keratoacanthoma-like features, may present with rapid growth and mimic inflammatory or infectious conditions [3].

The clinical spectrum of SCC is broad, ranging from actinic keratoses, considered intraepidermal precursors, to invasive lesions capable of deep tissue involvement and metastatic spread [1,4]. Although the majority of SCCs can be effectively managed with surgical excision or Mohs micrographic surgery, patient-specific factors such as age, comorbidities, and lesion characteristics may influence treatment decisions and outcomes [5]. Non-surgical modalities, including topical immunomodulators, may be considered in select low-risk or superficially invasive cases, particularly when patients are poor surgical candidates or prefer conservative treatment [6].

Here, we present a diagnostically complex case of keratoacanthoma-type invasive SCC in an elderly man with a history of actinic damage. Initially presumed infectious due to its rapid onset and appearance, the lesion was later confirmed histologically as well-differentiated SCC. The case highlights the importance of biopsy in atypical presentations and demonstrates the potential role of topical therapies under oncologic guidance in managing keratoacanthoma-like SCC.

## Case presentation

A 71-year-old man with a history of non-melanoma skin cancer and actinic keratoses presented for a routine full-body skin examination. A well-healed scar was noted on the left dorsal forearm from prior excision, along with hypertrophic erythematous papules with hyperkeratotic scale consistent with actinic keratoses. One lesion was routinely treated with liquid nitrogen cryotherapy.

**Figure 1:**
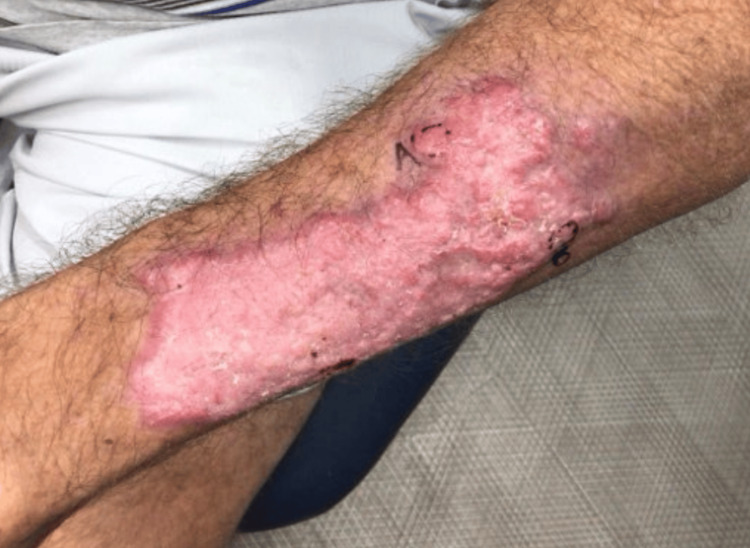
Pruritic, blistering, and scaly eruption localized to the left dorsal forearm

**Figure 2:**
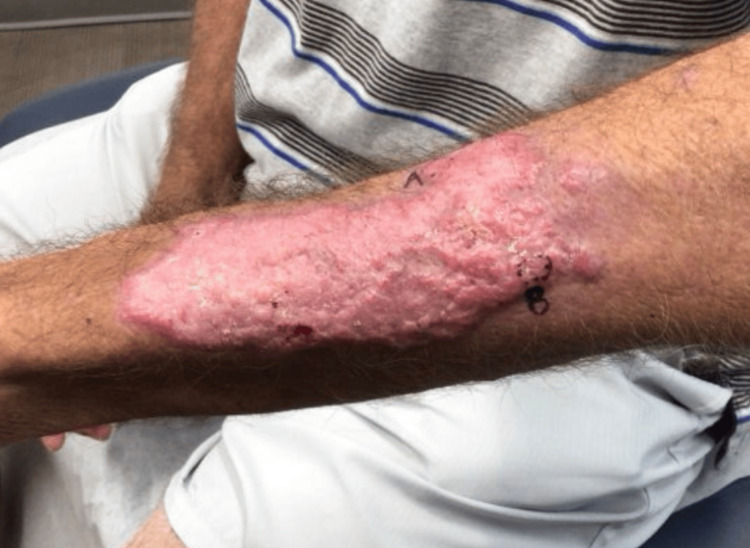
Pruritic, blistering, and scaly eruption localized to the left dorsal forearm

Approximately one month later, the patient returned with a new, pruritic, blistering, and scaly eruption localized to the left dorsal forearm (Figures 1, 2). He reported the lesion had developed three to four weeks prior. Examination revealed multiple erythematous macules and scaly papules on the proximal and distal radial aspects of the left forearm. A potassium hydroxide (KOH) prep demonstrated branching hyphae, and the patient was empirically started on oral terbinafine 250 mg daily for two weeks.

**Figure 3:**
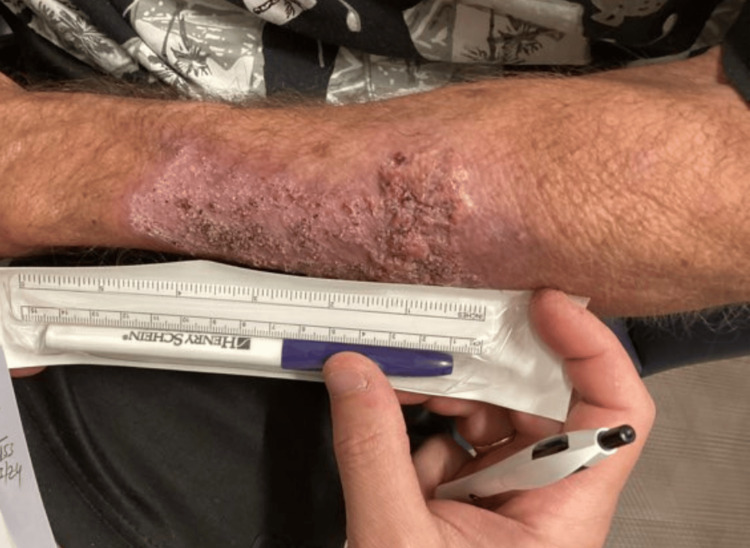
Improved lesion at follow-up measuring approximately 6.3 cm in diameter

**Figure 4:**
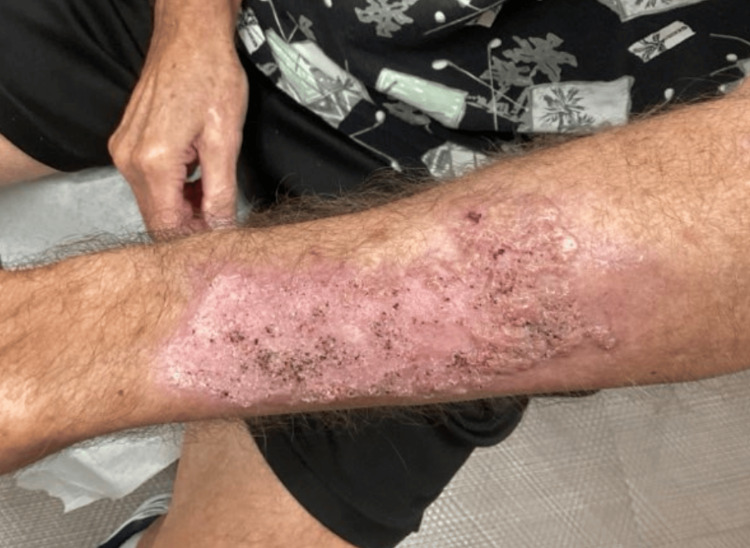
Improved lesion at follow-up measuring approximately 6.3 cm in diameter

Given the atypical appearance, two 4 mm punch biopsies were performed. One was taken from the proximal lesion and sent for histopathologic evaluation, while the other was taken from the distal lesion and submitted for bacterial, fungal, and mycobacterial cultures. Histopathology revealed well-differentiated invasive SCC of the keratoacanthoma type on the proximal forearm. The distal lesion was diagnosed as a keratoacanthoma. At the time of follow-up, the lesion had improved clinically, though it still measured approximately 6.3 cm in diameter (Figures 3, 4).

**Figure 5:**
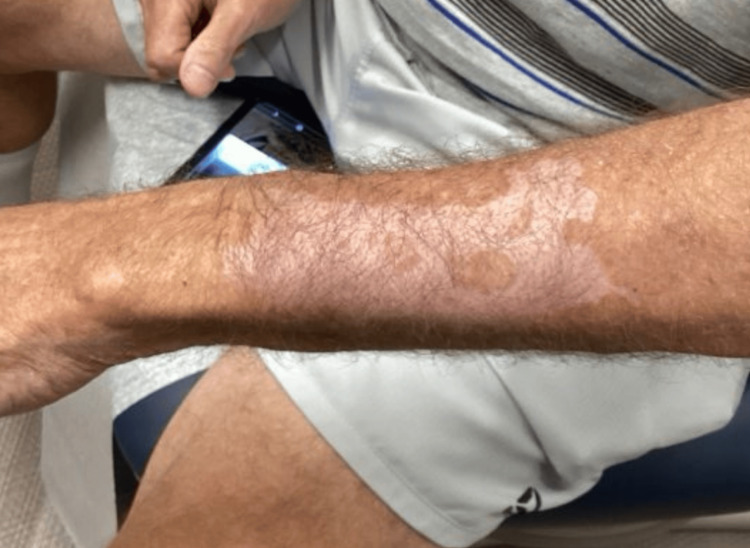
Clinically resolved lesion with only post-inflammatory hypopigmentation remaining

**Figure 6:**
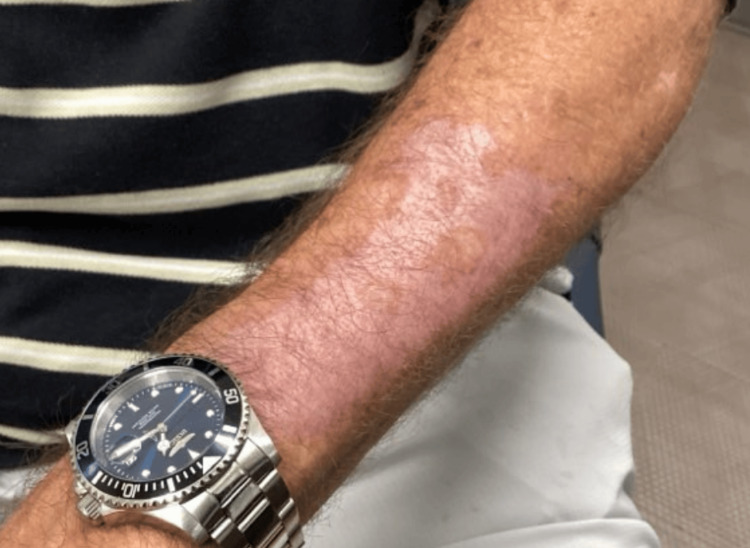
Clinically resolved lesion with only post-inflammatory hypopigmentation remaining

**Figure 7:**
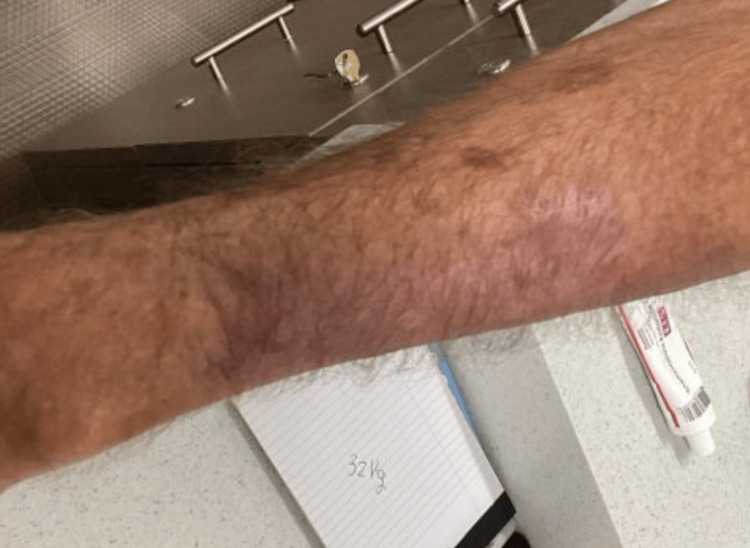
Clinically resolved lesion with only post-inflammatory hypopigmentation remaining

Due to its rapid growth and size, the patient was referred to a surgical oncologist. He was managed non-surgically with a topical regimen of imiquimod followed by triamcinolone, under close follow-up by oncology. A PET scan showed no evidence of metastasis. At follow-up several months later, the lesion had clinically resolved, with only post-inflammatory hypopigmentation remaining (Figures 5-7). No recurrence was noted.

Additional findings over the course of surveillance included stable actinic purpura, irritated seborrheic keratoses, idiopathic guttate hypomelanosis, and onychomycosis. Lesions were managed with cryotherapy or topical agents as appropriate. The patient declined full-body examinations at several visits but remained engaged in follow-up and was compliant with oncology-directed care. As of his last visit, the site of the SCC remained free of recurrence.

## Discussion

Cutaneous SCC with keratoacanthoma-like morphology presents a diagnostic challenge, particularly in elderly patients with rapid lesion development. While biopsy remains the gold standard for diagnosis, treatment can be complex when patients decline surgery or are poor surgical candidates. In our case, the patient opted for topical immunomodulators, an approach supported by emerging literature.

Several case reports document the use of topical imiquimod in managing invasive SCC. Dirschka et al. described an elderly patient with invasive SCC treated using 3.75% imiquimod cream, achieving both clinical and histological clearance without surgery [7]. Tiodorovic-Zivkovic et al. also reported successful resolution of two invasive SCCs in elderly patients using 5% imiquimod under oncologic supervision, further supporting topical immunotherapy in selected cases [8]. Additionally, Vidovic et al. combined topical 5% imiquimod with 5-fluorouracil (5-FU) in a renal transplant recipient with recurrent invasive SCC, resulting in complete remission under close monitoring [9]. These cases align well with our patient’s favorable response to topical therapy and oncology-guided care.

Keratoacanthomas share histologic features with SCC but often present a more rapid, crateriform appearance. Topical imiquimod has shown evidence-based success in treating keratoacanthomas. Jeon et al. reported four cases of keratoacanthoma treated with 5% imiquimod, achieving complete remission after nine to 11 weeks, suggesting its utility in lesions with KA morphology [10]. Similarly, Park et al. reviewed multiple keratoacanthoma cases treated with topical imiquimod, demonstrating consistent tumor regression over four to 11 weeks [11].

While the literature predominantly addresses superficial or well-differentiated lesions, off-label topical therapy for invasive SCC is gaining traction. A 2017 case series documented the use of 5% imiquimod for invasive SCC in elderly or immunosuppressed patients, noting good tolerance and disease regression under close follow-up [12]. Another series combined imiquimod with clobetasol or 5-FU, offering multi-modal topical therapy in non-surgical candidates [13].

However, immunomodulatory therapy is not risk-free. There are reports of paradoxical reactive keratoacanthomas emerging during imiquimod treatment, including eruptive SCC keratoacanthoma lesions in immunocompromised individuals, highlighting that clinicians should monitor for both treatment response and unexpected proliferation [14]. Most importantly, current consensus from geriatric oncology emphasizes tailoring SCC management to the patient's overall health, functional status, and preferences, not simply lesion size or aggression [15]. This patient’s advanced age, comorbidities, lesion characteristics, and active participation in oncology-directed topical management echo these core principles.

## Conclusions

This case illustrates the diagnostic and therapeutic complexity of keratoacanthoma-type SCC in an elderly patient. A rapidly developing forearm lesion initially presumed infectious was ultimately confirmed as invasive SCC, reinforcing the critical role of biopsy in atypical presentations. While surgical excision remains the standard of care, this case highlights how topical immunomodulation may offer a viable alternative in select patients with well-differentiated disease and appropriate oncology follow-up. As the aging population grows, clinicians must remain vigilant in evaluating new skin lesions and be prepared to tailor treatment strategies that align with both clinical guidelines and patient-centered care.

## References

[1] Que SK, Zwald FO, Schmults CD (2018). Cutaneous squamous cell carcinoma: Incidence, risk factors, diagnosis, and staging. J Am Acad Dermatol.

[2] Stratigos A, Garbe C, Lebbe C (2015). Diagnosis and treatment of invasive squamous cell carcinoma of the skin: European consensus-based interdisciplinary guideline. Eur J Cancer.

[3] Werner RN, Stockfleth E, Connolly SM (2015). Evidence- and consensus-based (S3) guidelines for the treatment of actinic keratosis - International League of Dermatological Societies in cooperation with the European Dermatology Forum - short version. J Eur Acad Dermatol Venereol.

[4] Alam M, Ratner D (2001). Cutaneous squamous-cell carcinoma. N Engl J Med.

[5] Wysong A (2023). Squamous-cell carcinoma of the skin. N Engl J Med.

[6] Rembielak A, Yau T, Akagunduz B (2023). Recommendations of the International Society of Geriatric Oncology on skin cancer management in older patients. J Geriatr Oncol.

[7] Dirschka T, Schmitz L, Bartha Á (2016). Clinical and histological resolution of invasive squamous cell carcinoma by topical imiquimod 3.75%: a case report. Eur J Dermatol.

[8] Tiodorovic-Zivkovic D, Zalaudek I, Longo C, De Pace B, Albertini G, Argenziano G (2012). Successful treatment of two invasive squamous cell carcinomas with topical 5% imiquimod cream in elderly patients. Eur J Dermatol.

[9] Vidovic D, Simms GA, Pasternak S (2021). Case report: combined intra-lesional IL-2 and topical imiquimod safely and effectively clears multi-focal, high grade cutaneous squamous cell cancer in a combined liver and kidney transplant patient. Front Immunol.

[10] Jeon HC, Choi M, Paik SH, Ahn CH, Park HS, Cho KH (2011). Treatment of keratoacanthoma with 5% imiquimod cream and review of the previous report. Ann Dermatol.

[11] Ko NY, Park JH, Son SW (2006). Treatment of keratoacanthoma with 5% imiquimod cream. Ann Dermatol.

[12] Tillman DK Jr, Carroll MT (2007). Topical imiquimod therapy for basal and squamous cell carcinomas: a clinical experience. Cutis.

[13] Fayne R, Nanda S, Nichols A, Shen J (2020). Combination topical chemotherapy for the treatment of an invasive cutaneous squamous cell carcinoma. J Drugs Dermatol.

[14] D'Addario S, Carrington PR (2006). Multiple keratoacanthomas as an untoward response to imiquimod therapy for actinic keratoses. Acta Derm Venereol.

[15] Nouri K, O'Connell C, Rivas MP (2003). Imiquimod for the treatment of Bowen's disease and invasive squamous cell carcinoma. J Drugs Dermatol.

